# Supplementary figures and images for: Phylomitogenomics bolsters the high-level classification of Demospongiae (phylum Porifera)
Source: PLoS One. 2023 Dec 4;18(12):e0287281. doi: 10.1371/journal.pone.0287281 (PMC10695373; doi:10.1371/journal.pone.0287281)

## Heteroscleromorpha

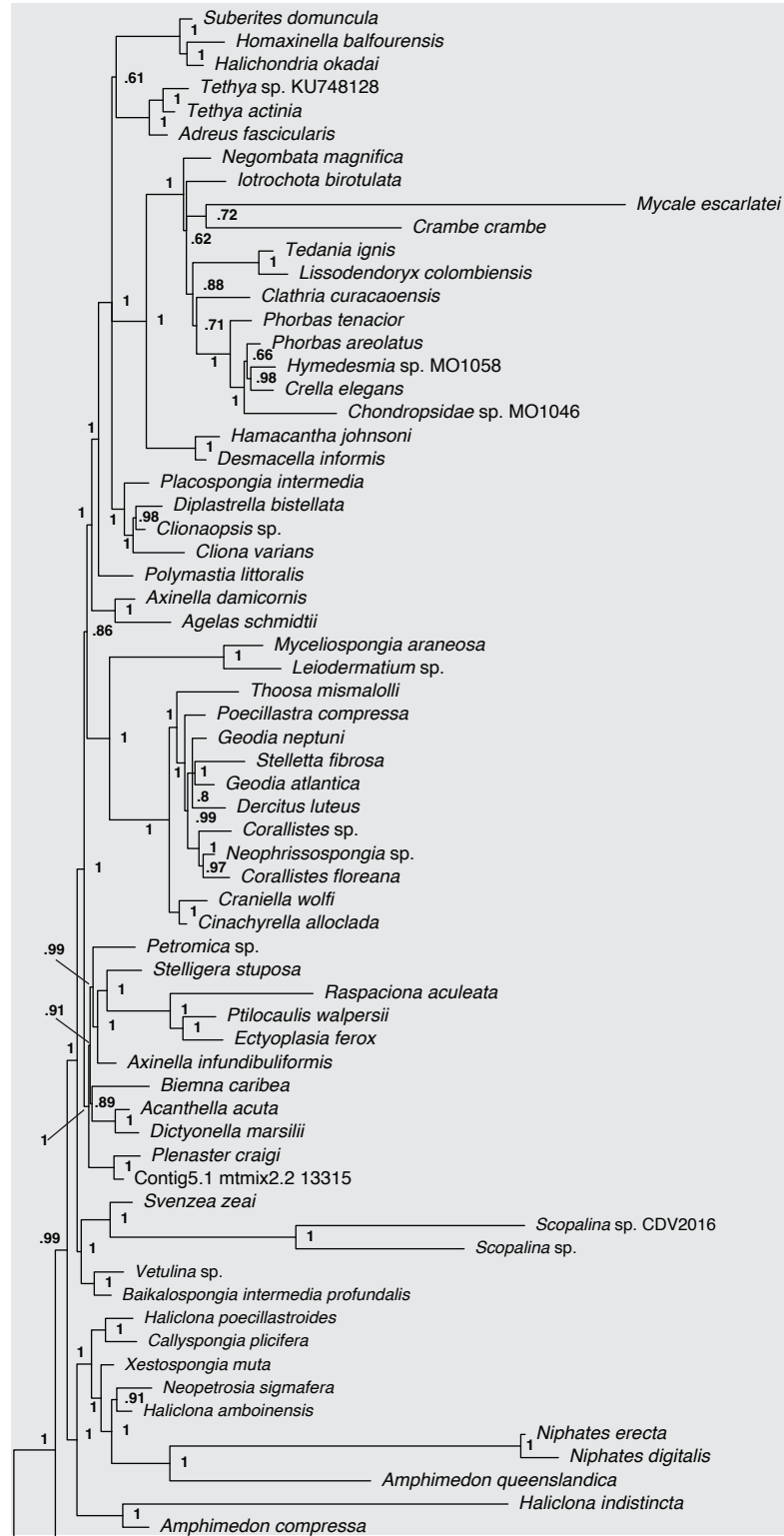

## Keratosa

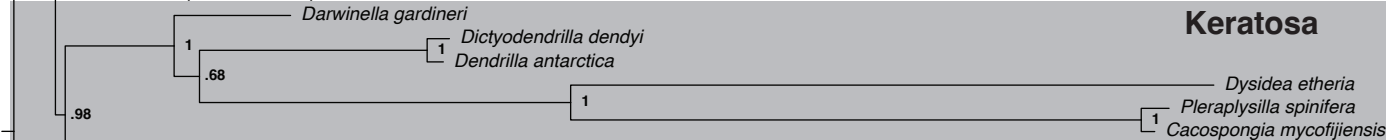

## Verongimorpha

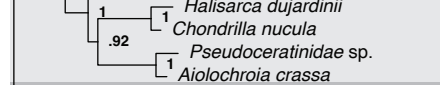

## Homoscleromorpha

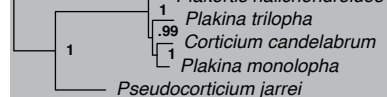

Supplement: S1 Fig — Posterior majority-rule tree was obtained from the analysis of concatenated mitochondrial amino acid sequences (3,633 positions) under the CAT+GTR+Γ model in the PhyloBayes-MPI program. Mitochondrial coding sequences from nine species of Homoscleromorpha were added to those of demosponges used for the analysis presented in Fig 2 and the resulting dataset was filtered with CD-Hit to remove sequences with >95% identity. The root was placed between Demospongiae and Homoscleromorpha. (PDF) [file pone.0287281.s001.pdf]

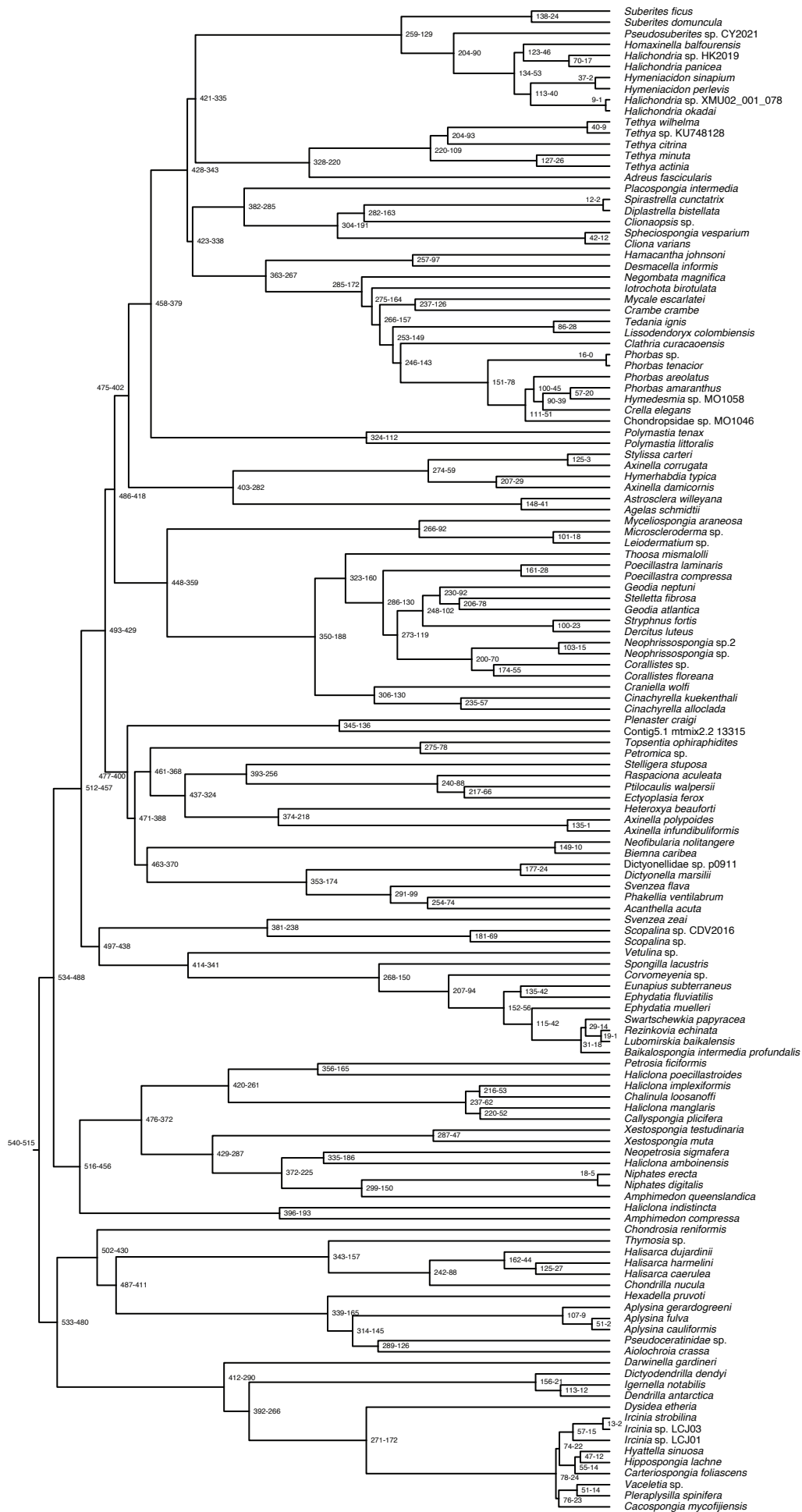

Supplement: S3 Fig — Numbers at internal nodes indicate their upper and lower age limits. (PDF) [file pone.0287281.s003.pdf]
