# Supplementary material for: Phylomitogenomics bolsters the high-level classification of Demospongiae (phylum Porifera)
Source: PLoS One. 2023 Dec 4;18(12):e0287281. doi: 10.1371/journal.pone.0287281 (PMC10695373; doi:10.1371/journal.pone.0287281)
Supplement: S2 Fig — Strict consensus of 12800 most parsimonious trees is shown with support values based on 200 bootstrap replicates. All analyses were conducted in TNT v. 1.5 using the “new technology” search options. (PDF) [file pone.0287281.s002.pdf]

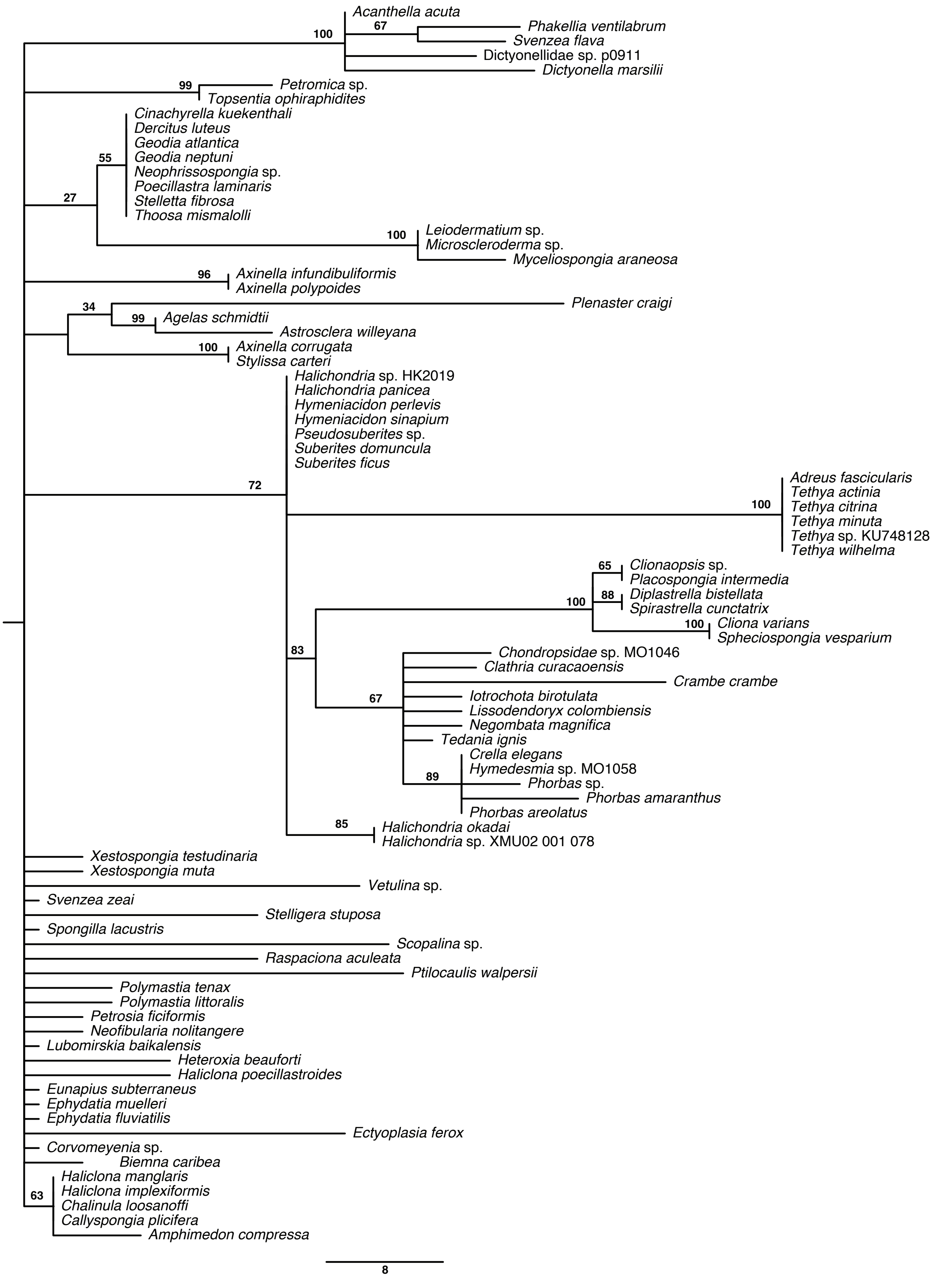

Acanthella acuta
Phakellia ventilabrum
Svenzea flava
Dictyonellidae sp. p0911
Dictyonella marsilii
Petromica sp.
Topsentia ophiraphidites
Cinachyrella kuekenthali
Dercitus luteus
Geodia atlantica
Geodia neptuni
Neophrissospongia sp.
Poecillastra laminaris
Stelletta fibrosa
Thoosa mismalolli
Leiodermatium sp.
Microscleroderma sp.
Myceliospongia araneosa
Axinella infundibuliformis
Axinella polypoides
Plenaster craigi
Agelas schmidtii
Astrosclera willeyana
Axinella corrugata
Stylissa carteri
Halichondria sp. HK2019
Halichondria panicea
Hymeniacidon perlevis
Hymeniacidon sinapium
Pseudosuberites sp.
Suberites domuncula
Suberites ficus
Adreus fascicularis
Tethya actinia
Tethya citrina
Tethya minuta
Tethya sp. KU748128
Tethya wilhelma
Clionaopsis sp.
Placospongia intermedia
Diplastrella bistellata
Spirastrella cunctatrix
Cliona varians
Spheciospongia vesparium
Chondropsidae sp. MO1046
Clathria curacaoensis
Crambe crambe
Iotrochota birotulata
Lissodendoryx colombiensis
Negombata magnifica
Tedania ignis
Crella elegans
Hymedesmia sp. MO1058
Phorbas sp.
Phorbas amaranthus
Phorbas areolatus
Halichondria okadai
Halichondria sp. XMU02 001 078
Xestospongia testudinaria
Xestospongia muta
Vetulina sp.
Svenzea zeai
Stelligera stuposa
Spongilla lacustris
Scopalina sp.
Raspaciona aculeata
Ptilocaulis walpersii
Polymastia tenax
Polymastia littoralis
Petrosia ficiformis
Neofibularia nolitangere
Lubomirskia baikalensis
Heteroxia beauforti
Haliclona poecillastroides
Eunapius subterraneus
Ephydatia muelleri
Ephydatia fluviatilis
Ectyoplasia ferox
Corvomeyenia sp.
Biemna caribea
Haliclona manglaris
Haliclona implexiformis
Chalinula loosanoffi
Callyspongia plicifera
Amphimedon compressa
100
67
99
55
27
100
96
34
99
100
72
100
65
100
88
100
83
67
89
85
63
8
