# Supplementary material for: Phylomitogenomics bolsters the high-level classification of Demospongiae (phylum Porifera)
Source: PLoS One. 2023 Dec 4;18(12):e0287281. doi: 10.1371/journal.pone.0287281 (PMC10695373; doi:10.1371/journal.pone.0287281)
Supplement: Attachment — Submitted filename: Response_to_Reviewers.pdf [file pone.0287281.s005.pdf]

Dear Dr. Lavrov,

Thank you for submitting your manuscript to PLOS ONE. After careful consideration, we feel that it has merit but does not fully meet PLOS ONE's publication criteria as it currently stands. Therefore, we invite you to submit a revised version of the manuscript that comprehensively addresses the points raised during the review process.

Please submit your revised manuscript by Oct 22 2023 11:59PM. If you will need more time than this to complete your revisions, please reply to this message or contact the journal office at plosone@plos.org. When you're ready to submit your revision, log on to https://www.editorialmanager.com/pone/ and select the 'Submissions Needing Revision' folder to locate your manuscript file.

Please include the following items when submitting your revised manuscript:

- A rebuttal letter that responds to each point raised by the academic editor and reviewer(s). You should upload this letter as a separate file labeled 'Response to Reviewers'.
- A marked-up copy of your manuscript that highlights changes made to the original version. You should upload this as a separate file labeled 'Revised Manuscript with Track Changes'.
- An unmarked version of your revised paper without tracked changes. You should upload this as a separate file labeled 'Manuscript'.

If you would like to make changes to your financial disclosure, please include your updated statement in your cover letter. Guidelines for resubmitting your figure files are available below the reviewer comments at the end of this letter.

If applicable, we recommend that you deposit your laboratory protocols in protocols.io to enhance the reproducibility of your results. Protocols.io assigns your protocol its own identifier (DOI) so that it can be cited independently in the future. For instructions see: https://journals.plos.org/plosone/s/submission-guidelines#loc-laboratory-protocols. Additionally, PLOS ONE offers an option for publishing peer-reviewed Lab Protocol articles, which describe protocols hosted on protocols.io. Read more information on sharing protocols at https://plos.org/protocols?utm_medium=editorial-email&utm_source=authorletters&utm_campaign=protocols.

We look forward to receiving your revised manuscript.
Kind regards,
Michael Schubert
Academic Editor
PLOS ONE
Journal requirements:
When submitting your revision, we need you to address these additional requirements.
1. Please ensure that your manuscript meets PLOS ONE's style requirements, including those for file naming. The PLOS ONE style templates can be found at https://journals.plos.org/plosone/s/file?id=wjVg/PLOSOne_formatting_sample_main_body.pdf and https://journals.plos.org/plosone/s/file?id=ba62/PLOSOne_formatting_sample_title_authors_affiliations.pdf

We used PLOS latex template (https://journals.plos.org/plosone/s/latex) to format the manuscript.

2. Please note that PLOS ONE has specific guidelines on code sharing for submissions in which author-generated code underpins the findings in the manuscript. In these cases, all author-generated code must be made available without restrictions upon publication of the work. Please review our guidelines at https://journals.plos.org/plosone/s/materials-and-software-sharing#loc-sharing-code and ensure that your code is shared in a way that follows best practice and facilitates reproducibility and reuse.

No author-generated code underpins the finding in the manuscript. All custom scripts that facilitate the analysis are available in the GitHub repository.

3. We note that the grant information you provided in the 'Funding Information' and 'Financial Disclosure' sections do not match.
When you resubmit, please ensure that you provide the correct grant numbers for the awards you received for your study in the 'Funding Information' section.

We corrected the information.

4. We note that you have included the phrase "data not shown" in your manuscript. Unfortunately, this does not meet our data sharing requirements. PLOS does not permit references to inaccessible data. We require that authors provide all relevant data within the paper, Supporting Information files, or in an acceptable, public repository. Please add a citation to support this phrase or upload the data that corresponds with these findings to a stable repository (such as Figshare or Dryad) and provide and URLs, DOIs, or accession numbers that may be used to access these data. Or, if the data are not a core part of the research being presented in your study, we ask that you remove the phrase that refers to these data.

We removed the phrase as requested.

5. Please include your full ethics statement in the 'Methods' section of your manuscript file. In your statement, please include the full name of the IRB or ethics committee who approved or waived your study, as well as whether or not you obtained informed written or verbal consent. If consent was waived for your study, please include this information in your statement as well.
[Note: HTML markup is below. Please do not edit.]

The ethical approval was not needed for this study because sponges are invertebrate animals without nervous system.

Reviewers' comments:

Reviewer's Responses to Questions
Comments to the Author

1. Is the manuscript technically sound, and do the data support the conclusions?

The manuscript must describe a technically sound piece of scientific research with data that supports the conclusions. Experiments must have been conducted rigorously, with appropriate controls, replication, and sample sizes. The conclusions must be drawn appropriately based on the data presented.
Reviewer #1: Yes
Reviewer #2: Yes
Reviewer #3: Yes

---

2. Has the statistical analysis been performed appropriately and rigorously?
Reviewer #1: Yes
Reviewer #2: Yes
Reviewer #3: Yes

---

3. Have the authors made all data underlying the findings in their manuscript fully available?

The PLOS Data policy requires authors to make all data underlying the findings described in their manuscript fully available without restriction, with rare exception (please refer to the Data Availability Statement in the manuscript PDF file). The data should be provided as part of the manuscript or its supporting information, or deposited to a public repository. For example, in addition to summary statistics, the data points behind means, medians and variance measures should be available. If there are restrictions on publicly sharing data—e.g. participant privacy or use of data from a third party—those must be specified.
Reviewer #1: Yes
Reviewer #2: Yes
Reviewer #3: Yes

---

4. Is the manuscript presented in an intelligible fashion and written in standard English?

PLOS ONE does not copyedit accepted manuscripts, so the language in submitted articles must be clear, correct, and unambiguous. Any typographical or grammatical errors should be corrected at revision, so please note any specific errors here.
Reviewer #1: Yes
Reviewer #2: Yes
Reviewer #3: Yes

---

5. Review Comments to the Author

Please use the space provided to explain your answers to the questions above. You may also include additional comments for the author, including concerns about dual publication, research ethics, or publication ethics. (Please upload your review as an attachment if it exceeds 20,000 characters)

Reviewer #1: I congratulate the authors on getting this huge dataset together and writing it up! I'd also like to thank them for submitting such a polished version for review, as it makes my job easy. The discussion, especially, was very nice. I love the authors honest discussions about doubt and uncertainty regarding fossil calibration. Great work!

We thank the reviewer for the kind words and constructive comments!

The dataset presented here, and its analysis, are undoubtedly valuable to the sponge community. The raw data will be used by many, and the tree will be thought provoking for a wide swath of evolutionary biologists. This value is also increased by the recent papers by Schuster et al. and Plese et al. that require correction, as pointed out (fairly) by the authors in the discussion. I would suggest the manuscript be accepted with only minor revisions required. My comments are very minor.

One point that struck me, however, was the omission of the recent paper by Pankey et al. 2022: https://www.nature.com/articles/s41559-022-01712-3 (and for the record: I'm not an author on that paper). The Pankey paper has time-calibrated phylogenies based on multiple loci, and is thus the most up-to-date and large-scale phylogeny available. The authors need to cite the work, and compare their results to Pankey. I think, for the most part, the results are very congruent -- even in terms of estimate divergence times, amazingly! -- and the papers will support each other very well (and add support to Lavrov et al.'s claims that the Plese & Schuster results are erroneous). The one possible exception might be that the established orders appear to be more problematic with the larger species sampling of Pankey -- though it is hard to tell how much data is available for any given species in their analysis, and they discuss their phylogenetic results in much less detail than Lavrov (indeed, barely at all!).

We thank the reviewer for bringing this article to our attention. We added some discussion (and citation) of this article.

Additional, exceedingly minor points:

I believe one of the author's names is misspelled.
We fixed the error.

Intro: line 52: you mean former not latter, right?
We meant latter (i.e., testing specific hypotheses).

You list voucher numbers in table 1, but it is unclear if these are actually archived in a permanent collection (and I really hope they are, since morphological data supporting the species IDs is not provided here, and some could be misidentified!). The authors need to explain what museum collections each of these numbers refers to, as no one can find " 28-V-91-2-001", for example, without knowing where to look. If some of these are vouchered in the author's labs instead of museums, I highly encourage the authors to voucher them in a more stable place.

Thank you for this comment! We added information about vouchers and also links that can be used to obtain collection information for the specimens.

The Plenaster species name is misspelled in the time-calibrated tree, I think
We checked the spelling.

Fig 4 caption mentions superorders...? Maybe proposed superorders? I don't know about any designated superorders.
The term was left accidentally from the previous version of the paper. We changed it to "clades" (as in the rest of the ms.)

Is fig S1 referenced in the paper? If it is, I missed where.
It is referenced within the "Phylogenetic analyses based on a supermatrix of inferred amino acid sequences" section, but the reference was not linked to the figure. We fixed the link.

Reviewer #2: I congratulate the authors on this long awaited fine piece of work. I found no conceptual issues to remark upon, but I confess I am not proficient on the details of the analyses undertaken. I can feel the underlying anxiety of wanting to turn big pieces of the current classification upside down, but authors were correctly conservative in this respect, which is expected as long as the Linnean typological classification rules our relation with the diversity of life. Said all this, please check the attached revised MS carfully for a series of typos highlighted. Also, I see a need for a carefull review of the references too, as many are abbreviated, while others are not.

We thank the reviewer for the kind words and careful reading of the ms! We corrected the typos and fixed all the issues with the references.

Reviewer #3: The manuscript by Lavrov and collaborators presents very interesting information regarding the systematic position of major demosponge orders recovered by the use of mitochondrial genomes, which is fundamental for understanding Porifera evolution. The authors have done a fantastic job sampling rare species and orders, and assembling a great number of new mitochondrial genomes, which are fundamental to understand sponge phylogeny. I think the manuscript deserves publication after major revision though, especially because there are some omissions of important results that should be discussed

in depth. Also, figure readability is crucial to make science accessible, and I would suggest improving the quality of the figures presented here.

We thank the reviewer for the kind words and useful comments on the ms.

Introduction

There seems to be an incongruence in the number of datasets sequenced and assembled: 64 sequenced and 6 assembled from existing data in the introduction, while it says 59 sequenced and 5 assembled from existing datasets. Please ensure these numbers are correct in the manuscript.

The number are correct as explained in the first paragraph of the Materials and Methods. 64 species in the introduction includes both complete and partial sequences.

Methods

I am afraid that the Table 1 provided is not sufficient to comply with the current standard metadata details necessary for reproducibility and open science. Please provide accurate location details, with longitude and latitude at least.

We modified the description of Table 1 to provide references to the permanent collection where the vouchers are deposited. These permanent collections contain additional data about specimens including longitude and latitude of the collection sites, when available. We also provided additional information about the sources of samples in Materials and methods.

In addition, there is one species coded as Unknown sp. Although I understand the importance of sequencing, the inclusion of completely undetermined species in phylogenetic trees with such relevance for sponge systematics should be avoided. Please provide some better taxonomic identification of the sponge, which I believe would not be extremely difficult given that among the authors are some of the best sponge taxonomists of the field.

The unknown species came as a contamination in one of the samples used for the analysis. We do not have any other data for this species, but given its important phylogenetic position, we included it in the analysis.

Please provide details for MAFFT alignment parameters.

We added the alignment parameters.

Mitochondrial genes are highly conserved across demosponges in general, so it seems unnecessary to use Gblocks to produce alignments. What was the rationale behind this? Did the authors try a non-gblocked alignment to compare the convergence?

Several species in the dataset had insertions in the coding sequences. The Gblocks was primarily used to remove regions with such insertions. Removing such regions is a standard practice in phylogenetic analysis.

Results

The finding of multi-chromosomic mitochondrial genomes in demo sponges is highly important and novel, and deserved more attention. There is not sufficient information to understand the specific features of the mt genome of Mycale escalatei. I understand this would be in line with other preliminary data, but

since that is not provided I suggest discussing here other possibilities for the lack of circularity (incompleteness, repetitive sequences, etc).

Unfortunately, we do not have any specific information about mt-genome organization in *Mycale escalatei* beyond what was reported in the paper. Because the main focus of this work is on phylogeny and systematics rather than mt-genome organization, there is no reason to include any speculations on it.

No information is provided for the position of the introns in the mt genes because it says they are being analysed in a separate study. Perhaps the phylogenetic affinities of such introns can be left for this separate study, but if the presence of introns is reported here, I would strongly encourage the authors to provide details somewhere for their positions.

The position of introns has no bearing on phylogeny or classification of Demospongiae. Therefore, we removed this information from the current version of the ms. However, their positions were reported in the previous version of this paper deposited in BioRxiv: https://doi.org/10.1101/793372. We added them to this version of the paper and leave it to the discretion of the Editor whether we should keep them or not.

Discussion
Lines 395-399: The recovery of a clade formed by Vetulina and Spongillida was also previously found by Plese et al. 2021.

While a clade formed by *Vetulina* and Spongillida is indeed recovered in Plese et al. 2021 study, their study did not add any new data relevant to this clade that were not present in the Schuster et al. (2018) paper. Therefore, we cited the earlier study instead. Furthermore, we cited the Redmond et al. (2013) study that first reported these relationships.

These lack of discussion with Plese et al. 2021 seems to be pervasive along the discussion, where it is only cited to discuss the unconventional relationships found there, which the authors claim that are likely to be found because unfortunate selection of outgroups. lthough I don't particularly agree with the view of the authors regarding these unfortunate outgroups, this could very well have been the case. However, that does not invalidate the entire results of Schuster et al. and Plese et al., which in turn found many of the same relationships found in the present study. One may wonder if there was a deliberate intention of not discussing the similar results found by these studies. For instance, the authors highlight the "unexpected" phylogenetic position of Axinella damicornis, although that was already placed within Agelasida by Plese et al. 2021. I would suggest discussing fairly and honestly the results of other researchers, giving credit where it is deserved, because it does not diminish the enormous relevance and importance of the present study.

The original version of the present paper was published as a pre-print back in 2019 (https://doi.org/10.1101/793372) and was cited by Plese et al. (2021). While five sequences from the Plese et al. (2021) were added to the dataset utilized in the current version of the ms., most of them were partial sequences belonging to the genera already represented in the tree. The addition of these sequences did not alter previously inferred phylogenetic relationships and therefore these data are not discussed. However, we tried to explain incongruences found among the studies.

The finding that Axinella is not a polyphyletic genus was reported by Gazave et al. back in 2010, was found in our analysis reported in 2019 and is not discussed further in the Plese et al. paper (the only reference to *A. damicornis* I could find is in the following sentence: "The following clade

groups together well-known agelasids (*Agelas schmidti*, *Cymbaxinella damicornis*, and *C. corrugata*) with *Stylissa carteri.*"

The main figure of the paper (Figure 2) is extremely difficult to follow and in my opinion, the quality is not sufficient for publication. Same for Figure 3. I suggest editing the figure to make the tree more readable and provide a summary tree with the relationships of the orders.

We agree that the versions of the figures included in the PDF file can be difficult to read. We think that this is, in part, is due to the relatively low resolution of the file. All the figures at high resolution are available in our online resource (https://lavrovlab.github.io/Demosponge-phylogeny/), which includes an option to view and manipulate the trees in iTol.

Other minor comments:

The name of the last author is not spelled correctly.

We fixed this typo.
